# Identifying gaps in HIV policy and practice along the HIV care continuum: evidence from a national policy review and health facility surveys in urban and rural Kenya

**DOI:** 10.1093/heapol/czx091

**Published:** 2017-07-28

**Authors:** Caoimhe Cawley, Ellen McRobie, Samuel Oti, Brian Njamwea, Amek Nyaguara, Frank Odhiambo, Fredrick Otieno, Muthoni Njage, Tara Shoham, Kathryn Church, Paul Mee, Jim Todd, Basia Zaba, Georges Reniers, Alison Wringe

**Affiliations:** 1 Department of Population Health, London School of Hygiene & Tropical Medicine, London, UK; 2 Department of Infectious Disease Epidemiology, Imperial College London, London, UK; 3 African Population and Health Research Centre, P.O. Box 10787-00100, Nairobi, Kenya; 4 KEMRI/CDC Center for Global Health Research (CGHR), P.O. Box 1578, Kisumu, Kenya; 5 Public Health Consultant, P.O. Box 42614-00100, Nairobi, Kenya

**Keywords:** Policy implementation, HIV policy, HIV care continuum, Kenya

## Abstract

The last decade has seen rapid evolution in guidance from the WHO concerning the provision of HIV services along the diagnosis-to-treatment continuum, but the extent to which these recommendations are adopted as national policies in Kenya, and subsequently implemented in health facilities, is not well understood. Identifying gaps in policy coverage and implementation is important for highlighting areas for improving service delivery, leading to better health outcomes. We compared WHO guidance with national policies for HIV testing and counselling, prevention of mother-to-child transmission, HIV treatment and retention in care. We then investigated implementation of these national policies in health facilities in one rural (Kisumu) and one urban (Nairobi) sites in Kenya. Implementation was documented using structured questionnaires that were administered to in-charge staff at 10 health facilities in Nairobi and 34 in Kisumu. Policies were defined as widely implemented if they were reported to occur in > 70% facilities, partially implemented if reported to occur in 30–70% facilities, and having limited implementation if reported to occur in < 30% facilities. Overall, Kenyan national HIV care and treatment policies were well aligned with WHO guidance. Policies promoting access to treatment and retention in care were widely implemented, but there was partial or limited implementation of several policies promoting access to HIV testing, and the more recent policy of Option B+ for HIV-positive pregnant women. Efforts are needed to improve implementation of policies designed to increase rates of diagnosis, thus facilitating entry into HIV care, if morbidity and mortality burdens are to be further reduced in Kenya, and as the country moves towards universal access to antiretroviral therapy.

Key Messages

- Kenyan national policies on HIV diagnosis, care and treatment aligned closely with recommendations from the World Health Organization.
- In a 2013/2014 survey of rural and urban health facilities, most Kenyan national policies relating to access to ART and retention in care were reported as widely implemented by facility managers, with the exception of Option B+ for pregnant women. However, several policies relating to HIV testing were reported to have limited implementation in both settings.
- Implementation of policies designed to facilitate diagnosis of people living with HIV was weak, and efforts are needed to support Kenya to reach its 90-90-90 targets and move towards elimination of HIV by 2030.

## Introduction

Over the last 10–15 years, the provision and uptake of HIV testing services has increased dramatically in sub-Saharan Africa, as has the number of people living with HIV (PLHIV) who are receiving antiretroviral therapy (ART). Despite this, rates of HIV testing remain sub-optimal in many countries (Venkatesh *et al.* 2011; Baggaley *et al.* 2012; Cawley *et al.* 2013). In addition, data on viral load monitoring among treated patients are sparse, yet viral load suppression is important in terms of not only individual patient outcomes but also ‘treatment as prevention’ and meeting UNAIDS ‘90-90-90’ targets (UNAIDS 2014b). Furthermore, higher rates of mortality persist among PLHIV compared with non-infected men and women (Reniers *et al.* 2014), largely due to high rates of attrition across the HIV care continuum before long-term adherence to treatment (Rosen and Fox 2011; Kilmarx and Mutasa-Apollo 2012; Kranzer *et al.* 2012; Bor *et al.* 2015). The WHO regularly releases clinical and programmatic guidance on the provision of HIV services, including recent recommendations for a universal ‘test and treat' approach (World Health Organisation 2015). However, the adoption of such recommendations within national HIV care and treatment policies is influenced by contextual factors operating within economic, political and social spheres (Hanney *et al.* 2003; Parkhurst and Lush 2004; Theobald et al. 2011; Edwards and Barker 2014), and multi-country studies have shown considerable heterogeneity in terms of which HIV care and treatment policies are adopted and when (Nelson *et al.* 2014; Kathryn Church *et al.* 2015).

In Kenya, the HIV epidemic can be described as generalised within the mainstream population, but also concentrated in most-at-risk populations (MARPs) such as men who have sex with men (MSM), commercial sex workers (CSW) and injecting drug users (IDU) (National AIDS Control Council 2014a,b). The National AIDS Control Council, the body responsible for coordinating the HIV response in Kenya, has developed a number of National Strategic Plans that detail priority interventions to tackle the HIV epidemic (National AIDS Control Council 2009; 2014b). These include interventions to improve the timely identification and linkage to care of PLHIV, as well as interventions to increase coverage of care and treatment, with a particular focus on reducing loss across the diagnosis-to-treatment continuum and on improving health outcomes. However, evidence on the translation of international guidelines and national-level HIV policies into practice at the health facility level is limited despite the fact that facility-level practices will influence health outcomes through various pathways including service access and coverage, quality of care, coordination of care and patient tracking, support to PLHIV and medical management (Mills *et al.* 2011; Wilson et al. 2014). Understanding HIV policy gaps relative to WHO recommendations, as well as contradictions between national level policy and practice, would be useful for policymakers concerned with improving HIV service delivery (Hirschhorn *et al.* 2007).

In this article, we compare the national HIV policy profile in Kenya with WHO policy guidance relating to HIV testing, prevention of mother-to-child transmission (PMTCT), and HIV care and treatment. We furthermore assess the extent to which Kenyan national HIV policies are implemented in health facilities serving the populations of an urban health and demographic surveillance site (HDSS) in Nairobi and a rural HDSS in Kisumu.

## Materials and methods

### Study setting

Nairobi is Kenya’s capital city with a fast-growing population, a large proportion of whom live below the poverty line in urban slums. The Nairobi Urban Health and Demographic Surveillance System (NUHDSS) was set up in 2002 in order to investigate the long-term social, economic and health consequences of urban residence among a population of about 120 000 people in two slum communities (Korogocho and Viwandani) (Beguy *et al.* 2015). HIV prevalence among adults aged 15–64 years in the Nairobi HDSS was most recently estimated at 12% in 2007 (Madise *et al.* 2012). The Kisumu Health and Demographic Surveillance System (KEMRI/CDC HDSS) is located in Rarieda, Siaya and Gem Districts in Nyanza province in Western Kenya and covers a combined population of about 220 000 people. Over 95% of the population are members of the Luo tribe and subsistence farming and fishing are the mainstay of the local economy (Odhiambo *et al.* 2012). HIV prevalence is among the highest in Kenya, estimated at 15% in 2013 (Kenya National AIDS and STI Control Programme 2014).

### Conceptual framework

**Figure 1.**
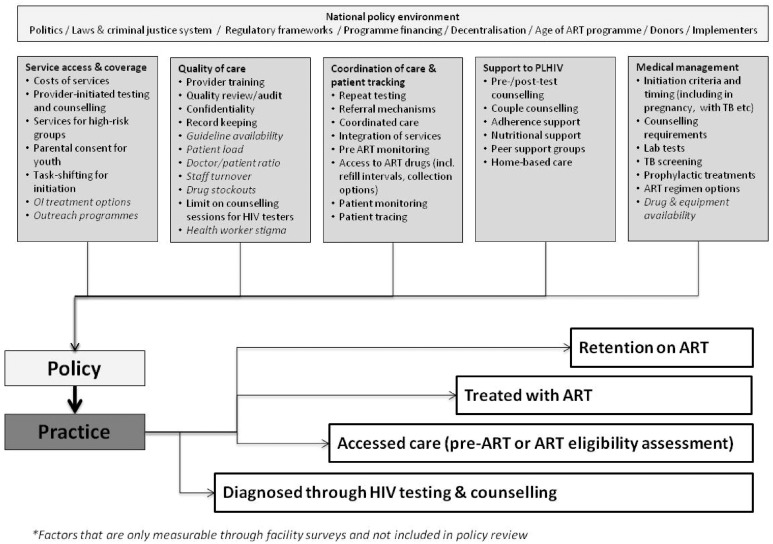
Conceptual framework identifying health policy and service factors influencing access to HIV services across the HIV care continuum.

As described previously, a conceptual framework identifying key HIV policy and programmatic factors that influence HIV-related adult mortality across the diagnosis-to-treatment continuum was developed by the ALPHA (Analysing Longitudinal and Population-based Data on HIV in Africa) Network (Kathryn Church *et al.* 2015) (Figure 1). This was based on findings from a review of the literature and expert consultation to identify health systems factors that influence access to HIV testing, treatment and retention in care.

### Policy review

As outlined previously (Kathryn Church *et al.* 2015), Kenyan national policy documents were retrieved through internet searches and email requests to national HIV bodies, and included if they met the following criteria: (1) nationally relevant (not clinic or district specific); (2) containing programmatic or clinical guidance on HIV testing and counselling (HTC), PMTCT or HIV care and treatment and (3) published between January 2003 and June 2013. Guideline documents produced by WHO relevant to criteria (2) and (3) were also retrieved. Additionally, three informal interviews were conducted with Kenyan policy makers and clinicians in order to supplement the national policy document review. In total 14 WHO reports and 14 Kenyan national policy documents included content that was relevant to the indicators assessed in this article (see Table 1). An Excel-based extraction tool was used to summarise information on policy content, source, year and policy changes over time. Policy indicators covering each of the five areas within the conceptual framework (service access and coverage, quality of care, coordination of care and patient tracking, support to PLHIV, and medical management, see Figure 1) were then generated. For each indicator, Kenyan policies were categorised as being explicit, implicit, partial (policy has caveats or exceptions), ambiguous (unclear whether policy exists, or policy conflicts with other policies) or absent.

**Table 1.** Summary of WHO and Kenyan national policies relating to HIV testing and treatment

**WHO policies**		**Year**
1	The Right to Know.	2003
2	Scaling up HIV Testing and Counselling Services: A Tool Kit for Programme Managers.	2004
3	UNAIDS/WHO Policy Statement on HIV Testing.	2004
4	Guidelines for Assurring the Accuracy and Reliability of HIV Rapid Testing: Applying a Quality System Approach.	2005
5	Anti-retroviral Therapy for HIV Infection in Adults and Adolescents: Recommendations for a Public Health Approach	2006
6	Antiretroviral Drugs for Treating Pregnant Women and Preventing HIV Infection In Infants: Towards Universal Access	2006
7	Guidance on Provider Initiated HIV Teating and Counselling in Health Facilities.	2007
8	Rapid advice: Anti-retroviral Therapy for HIV Infection in Adults and Adolescents.	2009
9	Deliver HIV Test Results and Messages for Re-testing and Counselling in Adults.	2010
10	Antiretroviral Drugs for Treating Pregnant Women and Preventing HIV Infection in Infants.	2010
11	Antiretroviral Therapy for HIV infection in Adults and Adolescents: Recommendations for a Public Health Approach	2010
12	Guidelines for Intensified Tuberculosis Case-finding and Isoniazid Preventive Therapy for People Living with HIV in Resource-constrained Settings	2011
13	Program Update: Use of Antiretroviral Drugs for Treating Pregnant Women and Preventing HIV Infection in Infants.	2012
14	Consolidated Guidelines on the Use of Anti-retroviral Drugs for Treating and Preventing HIV Infection	2013
**Kenya Policies**		**Year**
15	National ART Guidelines. NASCOP.	2005
16	National ART Guidelines. NASCOP.	2006
17	Presidential Declaration.	2006
18	Guidelines for HIV Testing and Counselling in Kenya. NASCOP.	2007
19	Kenya National Clinic Manual for ART Providers. NASCOP.	2007
20	Presidential Declaration.	2007
21	Guidelines for HIV Testing and Counselling in Kenya. NASCOP.	2008
22	National Guidelines for HIV/STI Programs for Sex Workers. MoPHS.	2010
23	National Recommendations for PMTCT of HIV, IYCF, and ART Therapy for Children, Adults, and Adolescents. MoPHS & MMS.	2010
24	National Quality Management Guidance Framework for HIV Testing and Counselling. NASCOP.	2010
25	Repeat and Re-testing Guidance. NASCOP.	2010
26	Guidelines for Antiretroviral Therapy in Kenya. NASCOP.	2011
27	Operational Manual for Community Based Testing for HIV Testing and Counselling. NASCOP.	2012
28	Guidelines for PMTCT of HIV/AIDS in Kenya. NASCOP.	2012

### Health facility surveys

A health facility survey questionnaire was developed as described previously (Church *et al.* 2017), drawing on the conceptual framework and other existing HIV health facility survey tools including the WHO Service Availability and Readiness Assessment surveys. The health facility surveys were carried out between October 2013 and May 2014. In Kisumu, all 34 health facilities located within the HDSS area were surveyed. In Nairobi, where there were no formal health facilities within the HDSS area itself, a convenience sample of 10 health facilities was taken to include different types or levels of health facility used by HDSS residents (5 were located on the outskirts of the HDSS area and the other 5 were located 2–5 km away).

The survey questionnaire captured data on health facility characteristics (e.g. staffing levels and client numbers), in addition to detailed information on HTC, PMTCT and ART services as they related to the five themes identified in the conceptual framework (Figure 1). The questionnaire was administered in English to the staff manager at each facility, or for some larger facilities, to the relevant staff manager within facility sub-units. Pharmacy records and logbooks were consulted to obtain accurate information on patient numbers, and drug and test-kit availability. Data were entered into MS SQL Server (Microsoft Corp, Redmond, USA) and exported for analysis using Stata11 (Stata Corp, TX, USA). Descriptive statistics were used to report on the provision of services according to pre-defined indicators, grouped by the five main areas identified in the conceptual framework as influencing the delivery of HIV services for adults.

### Policy implementation analysis

We assessed policy implementation in two different analyses. For both analyses, national policies were first classified as either explicit or not explicit (including policies which were implicit, partial, ambiguous or absent). In the first analysis, policy implementation was assessed in four HIV policy areas (testing, PMTCT, access to ART and retention in care), and analyses were broken down by HDSS site and size of facility (dispensaries and small health centres vs large health centres and hospitals) because it was hypothesized that levels of policy implementation may vary by these factors. Policy implementation was assessed as widespread if reported in practice in >70% of facilities (green shading in policy implantation analysis tables); partial if reported in 30–70% of facilities (orange shading in policy implantation analysis tables) or limited if reported in <30% of facilities (red shading in policy implantation analysis tables). The purpose of the second analysis was to assess overall policy implementation (at both small and large health facilities) across the five thematic areas identified in the conceptual framework (service access and coverage, quality of care, coordination of care and patient tracking, support to PLHIV, and medical management). In this analysis, policy implementation at the two HDSS sites was assessed for indicators with explicit policies only. This was in order to ease data interpretation, and also because indicators with non-explicit policies had already been assessed as part of the first analysis. A score of 1 was assigned for limited implementation of the policy in question, 2 for partial implementation and 3 for widespread implementation.

### Ethics

Ethical approval for the health facility surveys was granted by the authors’ institutions and ethics committees of the Kenya Medical Research Institute (KEMRI) and the London School of Hygiene and Tropical Medicine (LSHTM).

## Results

### Health facility characteristics

A summary of the characteristics of the facilities surveyed is shown in Table 2. In Nairobi, 4 of the 10 surveyed facilities were clinics while the remaining 6 were small or large hospitals. In Kisumu, 30 of 34 facilities surveyed were either dispensaries or small health centres, while the remaining four facilities were either small or large hospitals. All facilities at both sites provided HIV testing as well as PMTCT services, while 80% (8/10) in Nairobi and 94% (32/34) in Kisumu offered HIV care and treatment services. In terms of human resources indicators, the median numbers of healthcare workers per facility (as well as numbers of HIV testing clients and ART patients) were higher in Nairobi than in Kisumu. This is likely due to the higher proportion of larger facilities in Nairobi, and for the numbers of healthcare workers, the relative greater availability of medical personnel in the capital. Staff members at facilities in Kisumu had higher patient loads (mean numbers of HIV testing clients and ART patients per week) compared with staff members at facilities in Nairobi (Table 2). At both sites, the patient load or burden per staff member (number of weekly patients per clinician/nurse) was higher for ART provision than for HIV testing service provision (while national policies recommend a maximum number of HIV testing clients (*n* = 15) per healthcare worker per day, there is no such recommendation for ART clients).

**Table 2.** Summary characteristics of surveyed facilities in Kisumu and Nairobi

	Nairobi	Kisumu
Total number of clinics, *n*(%)	10 (100)	34 (100)
Type of facility		
Dispensary/small health centre	4 (40)	30 (88)
Large health centre/sub-district hospital	4 (40)	3 (9)
District/referral hospital	2 (20)	1 (3)
Management authority, *n* (%)		
Government	6 (60)	30 (88)
Faith-based organisation	3 (30)	3 (9)
Other NGO	1 (10)	0 (0)
Private-for-profit	0 (0)	1 (3)
HIV-related services, *n* (%)		
HIV testing	10 (100)	34 (100)
PMTCT	10 (100)	34 (100)
HIV care (incl. pre-ART)	8 (80)	32 (94)
HIV treatment	8 (80)	32 (94)
Human resources and patient load, median (range)		
No. of clinicians^a^	2.5 (0.0–8.0)	1 (0–34.5)
No. nurses/midwives	6.5 (0–18.0)	2 (0–62.0)
No. counsellors	2 (0–13.0)	0.3 (0–7.0)
No. HIV testing clients/week	78 (11.0–1237.0)	39 (0–206.0)
No. weekly HIV testing clients/staff^b^	1.3 (0.8–42)	3.7 (0–41.2)
Staff turnover^c^	11 (0–87.0)	0 (0–170.0)
Human resources for ART - clinics offering ART		
No. ART clients/week (median (range))	50 (35–141.0)	37 (0–154.0)
No. weekly ART clients/clinician or nurse (median (range))	4.8 (3.3–8.8)	11.7 (0–25.0)
Facilities with clinician, *n* (%)	7 (88)	25 (78)
Facilities with lab technician, *n* (%)	7 (88)	17 (53)

a Doctor, clinical officer, assistant medical officer.

b Nurse, midwife, nursing aide, counsellor or community outreach worker.

c No. staff left in past year/total staff (nurses, clinicians, aides, counsellors, outreach), as a percentage.

**Figure 2.**
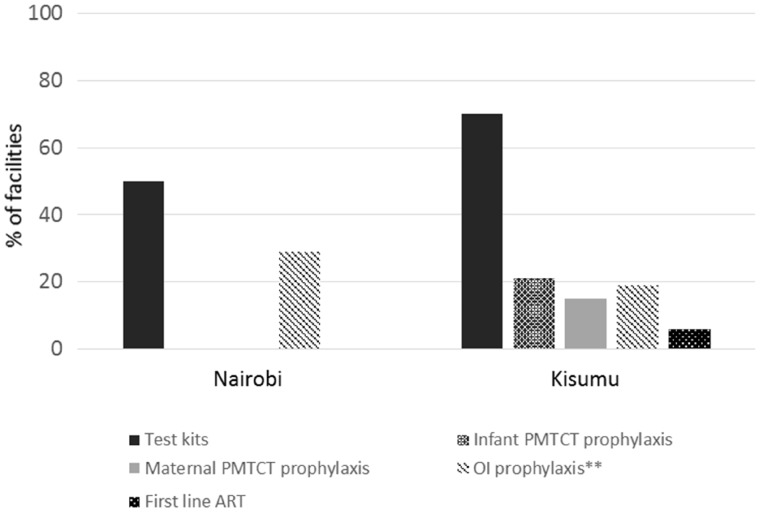
Percentage of facilities with frequent* stock-outs of HIV test-kits and treatment. *More than one in past year, or a stock-out lasting more than 2 weeks. **Opportunistic infection prophylaxis

A larger proportion of health facilities in Kisumu reported frequent stock-outs (defined as more than one stock-out in the past year, or a stock-out lasting >2 weeks) of HIV test kits, maternal or infant prophylaxis for PMTCT and first line ARVs compared with facilities in Nairobi (Figure 2). However, stock-outs of HIV test kits were common across both sites, at both small and large health facilities (50% of all facilities in Nairobi and 70% of all facilities in Kisumu reporting frequent stock-outs).

### Implementation of national HIV policies

#### HIV testing and counselling

Eleven indicators relating to HTC were investigated (Table 3). WHO policies existed for 10 of these indicators, of which 8 policies were explicit. Explicit Kenyan policies existed for all 11 indicators and most policies were reported as widely implemented across Nairobi and Kisumu (including both small and large facilities, 8/11 policies had wide coverage of implementation at both sites). A presidential declaration in 2006 stated that all HIV related services should be provided free of charge at government facilities, and accordingly 90% (9/10) of facilities in Nairobi and 100% (34/34) of facilities in Kisumu reported offering free HTC (Table 3) (even though only 60% and 88% of facilities were government-run in Nairobi and Kisumu, respectively). In addition, national quality management guidance stated that HIV-positive clients should be followed-up when referred for treatment, and 80% (8/10) and 97% (33/34) of facilities in Nairobi and Kisumu respectively reported having procedures to check whether HIV-positive patients had registered in care.

**Table 3.** Implementation of policies relating to HIV testing and counselling

	WHO policy (year of policy)^a^	Kenyan policy (year of policy)^a^	NAIROBI		KISUMU	
			Small facilities (*n* = 4)	Large facilities (*n* = 6)	Small facilities (*n* = 30)	Large facilities (*n* = 4)
Service access and coverage (%)						
Free testing at public facilities	**2003^1^**	**2006^17^**	**75**	**100**	**100**	**100**
PITC for ANC clients	**2004^3^**	**2007^18^**	**100**	**100**	**87**	**100**
HTC for high-risk groups	**2004^3^**	**2010^22^**	**75**	**67**	**20**	**75**
Quality of care (QoC) (%)						
Max 15 clients/day/counsellor	**None**	**2012^27^**	**75**	**67**	**53**	**25**
QoC reviews at least twice/year	**2005^4^**	**2010^24^**	**25**	**67**	**73**	**75**
Coordination of care and patient tracking (%)						
HIV- re-test every 6-12 months	**2007^7^**	**2010^25^**	**100**	**67**	**100**	**75**
Repeat testing in 3rd trimester	**2010^9^**	**2010^23^**	**100**	**83**	**43**	**75**
Check if HIV+ register at treatment site	**2004^2^**	**2010^24^**	**75**	**83**	**97**	**100**
Support to PLHIV (%)						
HTC pre-test counselling always given	**2003^1^**	**2008^21^**	**100**	**100**	**90**	**100**
HTC post-test counselling always given	**2003^1^**	**2008^21^**	**75**	**100**	**97**	**100**
Couples HTC offered	**2004^2^**	**2008^21^**	**100**	**83**	**67**	**100**

**KEY**

**WHO/Kenyan National Policies**
Explicit policy
No explicit policy (i.e. policy implicit, partial, ambiguous or absent)
**Kenya policy implementation analysis:**
Explicit policy, wide implementation
No explicit policy, wide implementation
Explicit policy, partial implementation
No explicit policy, partial implementation
Explicit policy, limited implementation
No explicit policy, limited implementation

a Superscript numbers relate to policy document reference number as listed in Table 1.

However, there was partial or limited implementation of some HTC indicators despite the existence of explicit national policy. For example, increasing HTC for high risk groups was highlighted as a priority area in the Kenya National AIDS Strategic Plan 2009/10-2012/13, and national guidelines state that vulnerable populations such as MSM, CSW and IDU should be provided with tailored and equitable access to HTC. Overall 70% of facilities in Nairobi reported offering HTC to high-risk groups (75% (3/4) of small facilities and 67% (4/6) of large facilities). However in Kisumu, only 20% (6/30) of small facilities reported offering HTC to high risk groups while 75% (3/4) of large facilities reported doing so (Table 2).

Other HTC policies which were explicit but had partial or limited implementation included a policy stating counsellors should receive no > 15 clients per day (implemented at 70% (7/10) of Nairobi clinics and 50% (14/34) of Kisumu clinics), quality of care reviews to be conducted twice per year (wide implementation in Kisumu [74% of facilities] but only partial implementation in Nairobi [50% of facilities]) and repeat testing for pregnant women in their third trimester (wide implementation in Nairobi [90% of facilities] but only partial implementation in Kisumu [47% of facilities]).

#### PMTCT

Seven indicators relating to PMTCT were investigated (Table 4). Explicit WHO policies existed for three of these indicators, while explicit Kenyan national policies existed for only two indicators. Despite this, widespread implementation of most indicators relating to PMTCT was reported. For example, 80% (8/10) and 91% (31/34) of facilities in Nairobi and Kisumu reported always providing PMTCT drugs for HIV-positive women who wanted or were likely to deliver at home despite the lack of an explicit national policy. One exception related to implementation of Option B+. While policy recommended that lifelong ART should be initiated in pregnant women as soon as possible, it was stated that provision of Option B+ should be limited to facilities with capacity to initiate this. Reported implementation of Option B+ was limited, with only 20% of Nairobi clinics (no small facilities and 33% (2/6) of large facilities) and 6% of Kisumu clinics (3% (1/30) of small facilities and 25% (1/4) of large facilities) reporting offering this as the standard of care for HIV-positive pregnant women (Table 4).

**Table 4.** Implementation of policies relating to PMTCT

	WHO policy (year of guideline)^a^	Kenyan policy (year of policy)^a^	NAIROBI		KISUMU	
			Small facilities (*n* = 4)	Large facilities (*n* = 6)	Small facilities (*n* = 30)	Large facilities (*n* = 4)
Service access and coverage (%)						
PMTCT^b^ available	**2010^10^**	**2012^28^**	**75**	**100**	**100**	**100**
Free PMTCT at public facilities	**Universal access**	**2006^17^**	**50**	**83**	**100**	**100**
Quality of care (QoC) (%)						
QoC reviews at least once/year	**None**	**None**	**75**	**100**	**93**	**100**
Coordination of care and patient tracking (%)						
PMTCT drugs always given for home delivery	**None**	**None**	**75**	**83**	**97**	**50**
Mothers referred for ART (during ANC/1 mo post-birth)	**2006^6^**	**2012^28^**	**100**	**100**	**83**	**75**
Check if mother registers for treatment	**None**	**None**	**75**	**100**	**100**	**100**
Medical management (%)						
Option B+ is standard	**2012^13^**	**2012^28^**	**0**	**33**	**3**	**25**

a Superscript numbers relate to policy document reference number as listed in Table 1.

b Prophylaxis or treatment for mother plus prophylaxis for baby.

#### HIV care and treatment

Eight indicators in relation to HIV care and treatment were assessed (Table 5). WHO policies existed for five of these indicators, of which three policies were explicit and two policies were partial or had caveats (left to countries to decide). At the Kenyan national level, policy adoption as well as implementation was reported as strong (six indicators with explicit policy and two without explicit policy). For six of eight indicators, implementation across both sites was reported to occur at 100% of facilities, including provision of free ART, allowing ART initiation by lower cadre health workers (clinical officers or nurses), being able to initiate ART within the ART clinic, conducting 6-monthly CD4 tests in the pre-ART phase, recording pre-ART visits on computers or patient registers, and enabling all patients with tuberculosis to initiate ART. Policy stated that laboratory tests such as renal or liver function tests were desirable but not a prerequisite for ART initiation. In practice, few facilities at either site reported initiating ART without any such tests (12.5% [1/8] of Nairobi clinics and 31% [10/32] of Kisumu clinics).

**Table 5.** Implementation of policies relating to HIV care and treatment

	WHO policy (year of guideline)^a^	Kenyan policy (year of policy)^a^	NAIROBI		KISUMU	
			Small facilities (*n* = 2)	Large facilities (*n* = 6)	Small facilities (*n* = 28)	Large facilities (*n* = 4)
Service access and coverage (%)						
Free ART at public facilities	**Universal access**	**2011^26^**	**100**	**100**	**100**	**100**
ART initiation by clinical officers or nurses	**None**	**2005^15^**	**100**	**100**	**100**	**100**
ART initiation in all ART clinic	**None**	**None**	**100**	**100**	**100**	**100**
Coordination of care and patient tracking (%)						
6 monthly CD4 testing in pre-ART phase	**None**	**2011^26^**	**100**	**100**	**100**	**100**
Pre ART visits recorded (e.g. computer, register, card)	**Left to country**	**2011^26^**	**100**	**100**	**100**	**100**
Medical management (%)						
Initiate ART at WHO Stage 3/4 or CD4≤350	**2009^8^**	**2011^26^**	**100**	**83**	**100**	**75**
Lab tests not required to start ART	**Recommended**	**2006^16^**	**50**	**0**	**32**	**25**
All patients with TB eligible for ART	**2009^8^**	**2007^19^**	**100**	**100**	**100**	**100**

a Superscript numbers relate to policy document reference number as listed in Table 1.

#### Retention in care

Fifteen indicators relating to retention in HIV care were assessed (Table 6). WHO policies existed for eight of these indicators, of which seven policies were explicit and one policy was implicit. At the Kenyan national level, explicit policies existed for 11 of the 15 indicators. Implementation of policies generally appeared stronger in Kisumu than Nairobi: all explicit policies were reported as widely implemented in Kisumu for both large and small facilities, whereas in Nairobi four explicit policies had only partial implementation coverage. Implementation was strong for two service indicators that had no explicit policy (for example, 100% [8/8] of Nairobi facilities and 94% [30/32] of Kisumu facilities reported providing home-based care for patients on ART). All facilities in both Nairobi and Kisumu provided at least one WHO recommended first line ART regimen according to 2010 guidelines (i.e. a TDF or AZT-based regimen—recalling that the health facility surveys were conducted in 2013/2014), while 88% (7/8) of Nairobi clinics and 75% (24/32) of Kisumu clinics reported offering at least four different first-line regimens. Policies relating to support to PLHIV were well implemented. For example 100% of facilities in both sites reported both peer support and nutritional support for patients on ART. Some indicators in relation to coordination of care were less well implemented in Nairobi facilities compared with Kisumu. For example, policy recommended that routine pill counts be conducted at every ART visit, but only 63% (5/8) of Nairobi facilities reported conducting these compared with 78% (25/32) of Kisumu facilities.

**Table 6.** Implementation of policies relating to retention in care

	WHO policy (year of guideline)^a^	Kenyan policy (year of policy)^a^	NAIROBI		KISUMU	
			Small facilities (*n* = 2)	Large facilities (*n* = 6)	Small facilities (*n* = 28)	Large facilities (*n* = 4)
Quality of care (%)						
Periodic checks on QoC at ART clinic	**2010^11^**	**2011^26^**	**100**	**100**	**96**	**100**
Coordination of care and patient tracking (%)						
6 monthly CD4 counts when on ART	**Not required**	**2005^15^**	**100**	**80**	**96**	**100**
3 monthly drug supplies once stable on ART	**None**	**2011^26^**	**50**	**67**	**79**	**75**
Routine pill counts once on ART?	**None**	**2005^15^**	**50**	**67**	**75**	**100**
Home visits following poor adherence	**None**	**None**	**0**	**17**	**32**	**50**
Home/phone contact following missed visit	**2013^14^**	**2011^26^**	**100**	**100**	**96**	**100**
Drugs collectable by designee	**None**	**None**	**50**	**100**	**100**	**100**
TB services integrated in facility	**2011^12^**	**2005^15^**	**100**	**100**	**100**	**100**
Support to PLHIV (%)						
Adherence counselling required	**None**	**2005^15^**	**100**	**100**	**100**	**100**
Peer support for people on ART	**None**	**2011^26^**	**100**	**100**	**100**	**100**
Nutritional support for people on ART	**2006^5^**	**2005^15^**	**100**	**100**	**100**	**100**
Home-based care for people on ART	**None**	**None**	**100**	**100**	**93**	**100**
Medical management (%)						
TB screen at every ART visit	**2011^12^**	**2005^15^**	**100**	**100**	**100**	**100**
WHO 1st line (2010) as standard	**2010^11^**	**2011^26^**	**100**	**100**	**100**	**100**
At least four first-line regimens available	**2006^5^**	**2011^26^**	**50**	**100**	**75**	**75**

a Superscript numbers relate to policy document reference number as listed in Table 1.

### Summarising policy implementation across the themes in the conceptual framework

**Figure 3.**
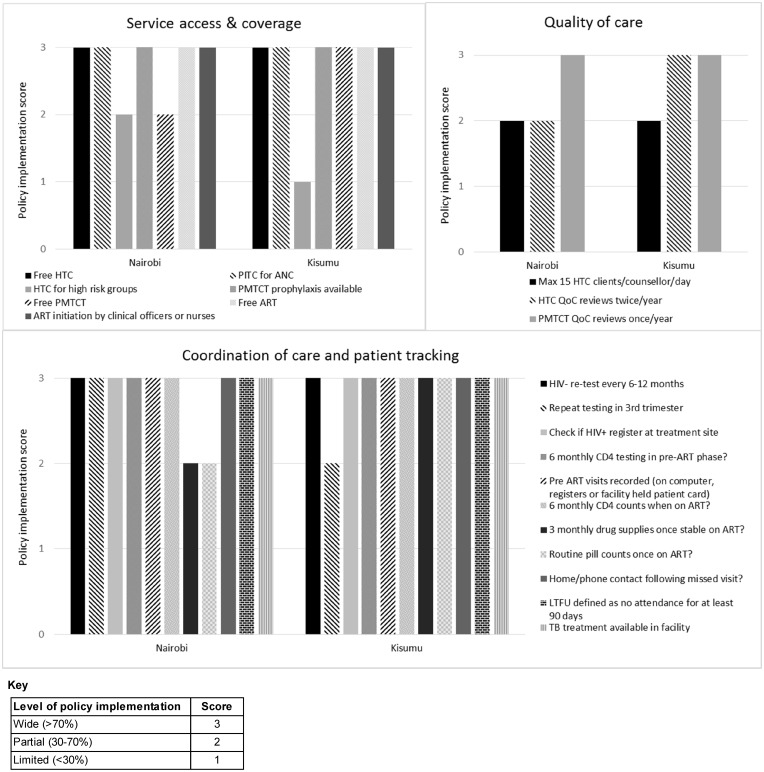
Policy implementation analysis by indicators influencing access to HIV services (indicators with explicit policy)

Across the five themes identified in the conceptual framework, there was generally widespread implementation (>70% of facilities implementing – score of 3) of indicators with explicit policies (Figure 3 shows results for themes ‘service access and coverage’, ‘quality of care’ and ‘coordination of care and patient tracking’. For themes ‘support to PLHIV’ and ‘medical management’, there was widespread implementation of all indicators with explicit policy). Themes with the largest proportions of indicators *without* explicit policy (indicators not shown in Figure 3) included (1) coordination of care and patient tracking and (2) medical management (non-explicit policies for 5/16 [31%] and 2/7 [29%] of indicators, respectively). Despite this, implementation of indicators relating to coordination of care was usually strong, even where policy was not explicit. For medical management, policy regarding Option B+ was not explicit, and implementation was limited.

## Discussion

We identified a close alignment between Kenyan national policies on HIV care and treatment and guidelines produced by the WHO, with a few instances of policy gaps in the areas of PMTCT, access to ART and retention in care. We also found that national policies were generally reported as widely implemented in Nairobi as well as Kisumu, and in both large and small facilities, with the exception of Option B+ (limited implementation) as well as limited of some explicit policies relating to HIV testing and counselling. The provision of HTC for high-risk groups was particularly poor, with only 9/34 (27%) facilities in Kisumu offering tailored HTC for CSW, MSM and IDUs. This contrasts with a call within the National Strategic Plan to increase access to HTC among MARPs in addition to the general population (National AIDS Control Council 2014b). It is worth noting however, that there was some level of support for this activity, which is not seen in other countries that do not specifically recognise these groups in their policy documents, such as Uganda (Kathryn Church *et al.* 2015). Epidemiological data indicating that infection is concentrated among MARPs in Kenya provide further support for the need to strengthen testing for these groups so that they may enter care at the earliest opportunity (Brodish *et al.* 2011; Muraguri *et al.* 2015; Musyoki *et al.* 2015). This is also of importance given the reported rise in injecting drug use across East Africa (Beckerleg *et al.* 2005).

There were gaps in the adoption of some policies relating to PMTCT (Option B+ as standard, PMTCT drugs always given for home deliveries, mothers referred for treatment during ANC or within one month after delivery, check if mother registers for treatment). Despite this, implementation was nevertheless reported as widespread at both sites for many indicators. This may reflect the influence of international organisations and the release of guidance from the WHO on PMTCT in 2013, or autonomous actions of facilities run by non-governmental organisations. There was a caveat to the Option B+ policy in Kenya, which stated this was recommended only at sites with the capacity to initiate this. Implementation of Option B+ was very limited (20% of clinics in Nairobi and 6% in Kisumu), which could be a marker of the perceived readiness of facilities to take on the additional long-term patient load and burden that would arise from complete adoption of Option B+. However given current WHO guidance, rapid expansion of Option B+ should be considered a programme priority in Kenya. An updated policy review and second round of facility surveys are being undertaken, and it will be interesting to see if implementation of Option B+ has changed or improved over time.

One policy influencing entry into care that was only widely implemented in Nairobi, but not Kisumu, was repeat testing of pregnant women in their third trimester. HIV testing and PMTCT are both key gateways to HIV care and ART, and should thus be a focus area for the Kenyan government if they hope to meet the 90-90-90 targets by 2020 (UNAIDS 2014b). Although facilities appeared to be offering many PMTCT services, despite lack of explicit policy, there is still a need for clear recommendations relating to testing of pregnant women and initiation onto ART from the Ministry of Health, in order to gain further traction and for revisions to allocation of resources. Antenatal and ART clinics will require significant investment and support in order to help them cope with increasing patient loads as a result of Option B+.

Policy adoption and implementation in relation to ART access and retention in care was strong in both rural and urban sites in this study. However in 2015 the WHO released guidance recommending ART initiation as soon as possible after diagnosis for all HIV-positive individuals, regardless of CD4 count, which is likely to put additional strain on HIV services (World Health Organisation 2015). At health facilities in both Kisumu and Nairobi, ART patient loads (number of weekly patients per clinician/nurse) were already higher at ART clinics than at HIV testing clinics, and so investments in human resources as well as task-shifting to lower cadre healthcare workers and/or use of alternative strategies for ART delivery will be required to support the move towards test and treat, if this is adopted as national level policy. The majority of health facilities at both sites required at least some lab tests before ART initiation (usually renal or liver function tests, or haemoglobin counts), and this may also prove challenging in terms of workload if test and treat strategies are adopted (Nachega *et al.* 2014). Another area of concern at both sites related to stock-outs of HIV test kits (frequent stock-outs reported at 70% and 50% of facilities in Kisumu and Nairobi respectivelyStock-outs of ART and other prophylactic drugs were less common. Further investigation at the sites would be needed to understand what factors enable smooth provision of HIV test kits and some medications over others. It is likely that the lower proportion of facilities reporting stock-outs in Nairobi compared with Kisumu is related to distance from central supply stores, however, reliable supply chains across the whole country and along the care continuum are required in order to meet 90-90-90 targets and maximise the effectiveness of HIV service provision.

The finding that policy implementation was widest in relation to treatment and retention in care, and poorer for HIV testing and PMTCT, contrasts with the situation in other settings. In Malawi, emphasis has been on implementation of policies to promote rapid initiation of ART, with weaker implementation of strategies to promote retention in care (e.g. adherence monitoring, home-based care) (Dasgupta *et al.* 2016). A similar finding has been reported for Uganda where policy adoption and implementation have been progressive for HIV testing, treatment, and PMTCT, but often lacking in relation to retention in care (McRobie *et al.* 2015). Further qualitative investigation might help to draw out the possible barriers or facilitating factors to policy adoption in different areas across different settings.

In terms of the themes identified in the conceptual framework, there was no single domain that stood out as particularly weak in terms of policy implementation across the different areas of service delivery that we investigated. For the themes relating to support to PLHIV and medical management, scores were high for all indicators with explicit policies. This is encouraging given the current nature of HIV as a chronic condition requiring long-term management and care.

There are some limitations that need to be considered when interpreting the findings from this study. First, health facility attributes and practices were reported by staff and may have been subject to reporting bias, resulting in a more positive picture of policy implementation than is the reality. Second, the health facilities surveyed were those serving the populations at the HDSS sites Nairobi and Kisumu, and were not selected to be representative of all HIV clinics in the country. Nevertheless, the clinics do not receive additional interventions or support as a result of the HDSS activities, and can therefore be considered as fairly typical of the services that are available to these populations.

## Conclusions

Targeting policy implementation gaps identified by this study, notably those relating to HIV testing and implementation of Option B+, would help Kenya to reach the ambitious 90-90-90 targets by 2020. Smaller health facilities are likely to require additional support to implement test and treat strategies once these are adopted as national level policy.
